# Refactoring the Genetic Code for Increased Evolvability

**DOI:** 10.1128/mBio.01654-17

**Published:** 2017-11-14

**Authors:** Gur Pines, James D. Winkler, Assaf Pines, Ryan T. Gill

**Affiliations:** a Renewable and Sustainable Energy Institute, University of Colorado Boulder, Boulder, Colorado, USA; b Department of Chemical and Biological Engineering, University of Colorado Boulder, Boulder, Colorado, USA; Korea Advanced Institute of Science and Technology

**Keywords:** evolution, genetic code, genome synthesis, saturation mutagenesis

## Abstract

The standard genetic code is robust to mutations during transcription and translation. Point mutations are likely to be synonymous or to preserve the chemical properties of the original amino acid. Saturation mutagenesis experiments suggest that in some cases the best-performing mutant requires replacement of more than a single nucleotide within a codon. These replacements are essentially inaccessible to common error-based laboratory engineering techniques that alter a single nucleotide per mutation event, due to the extreme rarity of adjacent mutations. In this theoretical study, we suggest a radical reordering of the genetic code that maximizes the mutagenic potential of single nucleotide replacements. We explore several possible genetic codes that allow a greater degree of accessibility to the mutational landscape and may result in a hyperevolvable organism that could serve as an ideal platform for directed evolution experiments. We then conclude by evaluating the challenges of constructing such recoded organisms and their potential applications within the field of synthetic biology.

## IMPORTANCE

The conservative nature of the genetic code prevents bioengineers from efficiently accessing the full mutational landscape of a gene via common error-prone methods. Here, we present two computational approaches to generate alternative genetic codes with increased accessibility. These new codes allow mutational transitions to a larger pool of amino acids and with a greater extent of chemical differences, based on a single nucleotide replacement within the codon, thus increasing evolvability both at the single-gene and at the genome levels. Given the widespread use of these techniques for strain and protein improvement, along with more fundamental evolutionary biology questions, the use of recoded organisms that maximize evolvability should significantly improve the efficiency of directed evolution, library generation, and fitness maximization.

## INTRODUCTION

**FIG 1:**
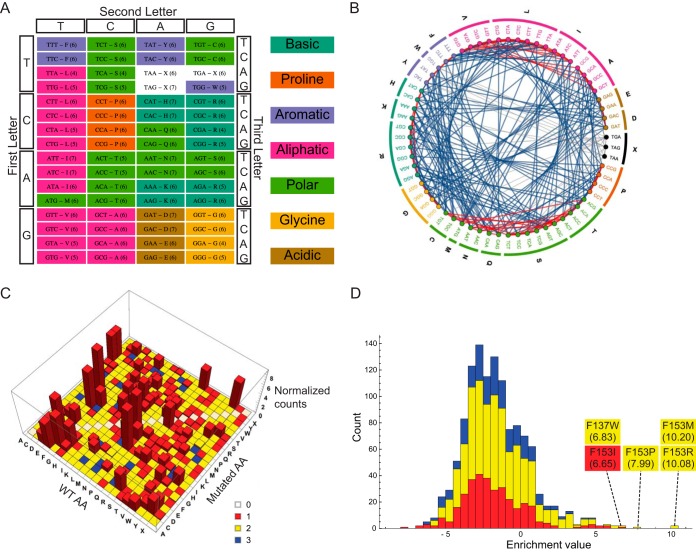
The properties of the standard genetic code. (A) The standard genetic code. Colors indicate the amino acid chemical classes (depicted on the right side of the chart). Stop codons are white and are denoted by X. Numbers in parentheses indicate the number of SNR-accessible unique amino acids for each codon. (B) The codon SNR accessibility plot of the standard code. All 64 codons are grouped according to their corresponding amino acids, which in turn are clustered by their chemical classes (colors are as for panel A). Edges connecting two codons indicate that these two codons are within an SNR distance. Edge colors correspond to whether the amino acid change is within the same chemical class (red) or between classes (blue). Edges connecting to stop codons are shaded gray. (C) Analysis of a previously reported database describing resistance mutations under a wide array of conditions (adapted from Winkler et al. 2016 [44]). The *x* and *y* axes correspond to the wild-type (WT) and mutated amino acids in resistance-conferring genes, respectively. The *z* axis represents normalized counts of the mutations. Colors indicate the minimal number of nucleotide replacements needed for the transition from WT amino acid to the mutated one. Note that the vast majority of mutations may be explained by a single nucleotide replacement within a codon. (D) A stacked view histogram of scanning saturation mutagenesis data from Garst et al. (32), showing the mutational fitness landscape following the incubation of a *folA* library with trimethoprim. Colors are as described for panel C. Note that the four most enriched mutants require two nucleotide replacements within the codon (enrichment values and mutation identity are indicated).

The deciphering of the genetic code has yielded many insights into its organization and evolution (1). Notably, the standard genetic code has been found to be robust to many single nucleotide replacements (SNRs), and similar codons code for amino acids with related properties (Fig. 1A) (2–5). Theories around the origin of the standard code organization focus on three main ideas: the first is the stereochemical theory, which claims that the current genetic code was formed by primordial interactions between codons (or anticodons) and amino acids (6). The second, termed the coevolution theory, points to the fact that amino acids sharing biosynthetic pathways are generally assigned to similar codons and suggests that the genetic code today reflects this coevolution (7–9). The third theory hypothesizes that the genetic code has been under selection to minimize deleterious changes in physicochemical properties caused by mutations and mistranslations (3, 10). A combination of all three theories is also a possibility (11). Regardless of the exact mechanism, the modern standard genetic code is indeed relatively robust to the effects induced by SNRs, and it buffers their effects (2, 12–14). As a result, a given amino acid can only be converted on average into 6.1 others (including the stop codon) without multiple nucleotide changes. While this property is favorable for buffering mutational effects in free-living organisms, it limits the effectiveness of mutagenesis techniques that rely on single nucleotide substitutions to alter coding sequences.

Saturation mutagenesis techniques provide full access to all amino acids by using degenerate codons that cover a comprehensive collection of amino acids (15–17). Such approaches frequently identify multiple nucleotide replacements (MNRs) within a codon to be superior to SNRs in a variety of contexts (16, 18–26). Recent technological advances in DNA synthesis and sequencing have enabled more detailed surveys, using complete systematic saturation mutagenesis of a gene and permitting the interrogation of the full mutational landscape at single-amino-acid resolution (27–30). Data from such experiments are still relatively rare but support the notion that the highest peaks in the mutational landscapes can require MNRs that are inaccessible in reasonable time frames when using only SNRs (31–33).

For investigations of the combinatorial space, saturation mutagenesis may be performed iteratively but requires *a priori* knowledge of sites of interest (34–36). Computational approaches intelligently select sites of interest, thus reducing the need to survey all possible mutations (37–39), but the risk of missing important sites still exists; the desired phenotype may consist of multiple unpredictable changes in genes and other genetic elements. As a result, adaptive laboratory evolution experiments are still the gold standard for identifying desired phenotypes when the underlying genotype-phenotype relationship is unclear. However, laboratory evolution cannot access the full amino acid repertoire, since the changes are primarily limited to SNRs that alter protein coding sequences and thus might not be able to access the genotypes at the global fitness maximum. While further improvements are feasible with further secondary mutations, the multiple rounds of diversity generation followed by selection slow the process of strain or protein improvement significantly (40–43).

Here, we suggest that several reorderings of the genetic code will enable significantly more comprehensive exploration of fitness landscapes and simplify the identification of beneficial mutations. We apply two computational approaches for the generation of such genetic codes, focused on increasing the numbers and chemical diversity of SNR-accessible amino acids. We discuss a range of alternative codes that have altered SNR accessibilities. Subsequently, we recode the set of essential *Escherichia coli* genes by using these generated codes to evaluate the high-level impact of genetic code refactoring. After looking at the properties of theoretical codes, we then discuss both the practical difficulties for implementing these hypothetical codes in a living organism and potential biotechnological applications for novel, highly evolvable genetic codes.

## RESULTS

### Amino acid accessibility.

At the amino acid level, the standard genetic code allows an SNR to convert a codon into alternative codons encoding 6.1 unique amino acids. The distribution is not wide, as both the standard deviation (0.69) and range (5 to 7 amino acids) are low as a result of the constraints placed on mutational accessibility due to the architecture of the trinucleotide genetic code. On average, 5.53 different chemical classes are accessible by the same mutations (see Materials and Methods for our amino acid classification source).

Figure 1A depicts the standard genetic code organization, with the amino acids colored according to their chemical classes and numbers in parentheses indicating the number of unique SNR-accessible amino acids. An additional manner of illustrating the SNR-accessible amino acids is shown in Fig. 1B. Here, the codons are ordered in a circle, grouped according to the encoded amino acids and classes. Edges connecting two codons represent SNR accessibilities, and edge colors indicate whether amino acid conversion is within (red) or between (blue) chemical classes. This plot highlights the notion that many SNR-accessible amino acids are chemically similar, as depicted by the multitude of red edges. Edge distributions of all genetic codes presented in this study are shown in Fig. S2 in the supplemental material.

To investigate the potential effect of mutation buffering that favors SNR-induced amino acid replacements, we analyzed a recently published collection of 2,679 amino acid changes generated by various random methods associated with diverse resistance phenotypes (44). The results showed that almost all mutations found could be explained by a single nucleotide replacement, with double and triple nucleotide replacements being required for at most 3.5% of all detected mutations (Fig. 1C). The number of nucleotide replacements required for inter-amino acid conversion and their observed frequency in the data are strongly anticorrelated (*P* < 10^−10^, Spearman test), comporting well with previous findings (33). If we take into account that this database is related to the mutational response to stress, which is known to be associated with an elevated mutation rate, this increases the significance of these findings even further (45–47). These findings highlight the confined space evolution is allowed to explore, since it is mostly limited to SNRs.

Recently, we reported a novel method for high-throughput, genome-wide, single-amino-acid-level genome editing in *E. coli* (32). A complete scanning saturation mutagenesis of the dihydrofolate reductase (DHFR) gene, *folA*, and incubation with the DHFR-specific inhibitor trimethoprim were performed to find resistant mutants. Multiple mutants were found to be enriched following trimethoprim treatment, including mutations at several sites that were previously reported (40, 48). However, many of those sites, including the mutants with the highest fitness values (Fig. 1D), had novel MNR mutations, since most experiments were previously done using SNR-based methods, such as directed evolution (40, 48). These results are in line with other findings, suggesting that MNRs are more effective at phenotype improvement than SNRs found by random mutagenesis methods (16). Other saturation mutagenesis libraries also show MNR superiority over SNRs under several adaptive conditions (27, 31–33). Taken together, these examples, along with the fundamentally conservative nature of the genetic code, support the hypothesis that sometimes a radical change in the amino acid characteristics is required for a drastic shift in the corresponding protein’s properties and that such changes are simply not available using SNRs alone.

### Generation of alternative genetic codes.

Having identified a critical factor limiting the effectiveness of current directed evolution tools, uncovering possible genetic changes that can reduce the buffering capacity of the standard genetic code is of interest. So far, *E. coli* has been engineered to lack the TAG stop codon, and a larger-scale ongoing effort to remove seven codons from the genome has been reported (49, 50). These efforts establish the conceptual feasibility of altering the code and producing a viable organism with a more “evolvable” code, if it can be designed and implemented successfully. One approach to generating new candidate codes is through the use of a genetic algorithm that evolves new codes according to user-defined criteria. Genetic algorithms have been used to optimize other properties of genetic codes in the past, typically focusing on robustness (51) or propagation of error to the protein level (52). The principal requirement for implementing a genetic algorithm is to define a fitness function F that can be maximized to select among a population of randomly modified codes to identify those encoding our desired characteristics (outlined above).

The first component, F_unique_, of our proposed fitness function is simply the average cardinality of the set of amino acids accessible by each codon within the genetic code using SNRs (equation 1), where num(*) represents the cardinality operator, AA(C) represents the amino acid encoded by codon C, *D*(X,Y) is a Hamming distance function, and *N* is the number of codons in the genetic code. The second component, F_ratio_, is calculated by first determining the number of times an SNR will convert any other amino acid into a given amino acid and then dividing the minimum and maximum values to obtain the ratio of interest for a code with M total amino acids (equation 2). Codes with more even distributions of codons between amino acids will have F_ratio_ values closer to 1. The final component of F is simply the number of nondistinct chemical classes accessible by SNRs, denoted as F_chem_ (equation 3). The final fitness function (equation 4) is then taken as the product of these components, normalized by their theoretical maximums to avoid inadvertently favoring improvement in a specific area over the others (see Materials and Methods).

However, one limitation of this approach is that there is no guarantee that the final ensemble of improved codes has reached the global fitness optimum, given the stochastic nature of the algorithm and extremely large number of possible valid codes. Calculation of F(C) scales poorly because accessibility between all pairs of amino acids must be computed and this becomes computationally limiting if the number of candidate codes per round of selection is large.

(1) $${\text{F}}_{\text{unique}}=\text{num}(\text{AA(}{C^{\prime }}_{i})\text{ where D}(\text{C},\;{C^{\prime }}_{i}\text{)}=1)/N$$

(2) $$F_{\text{ratio}}=\text{min}({\Sigma_{j}}^{N}\text{if AA}(C=>C^{\prime })==A_{i}\;1\text{ else }0)/\text{max}(..)\text{for i}=\left\{0,M\right\}$$

(3) $${\text{F}}_{\text{chem}}=\text{num}(\text{CHEM\_CLASS(AA(}{C^{\prime }}_{i}))\text{where D}(C,{C^{\prime }}_{i})=1)/N$$

(4) $$\text{F}={\text{F}}_{\text{unique}}/{\text{F}}_{\left(\text{unique},\;\max\right)}*{\text{F}}_{\text{ratio}}/{\text{F}}_{\left(\text{ratio},\;\max\right)}*{\text{F}}_{\text{chem}}/{\text{F}}_{\left(\text{chem},\;\max\right)}$$

**FIG 2:**
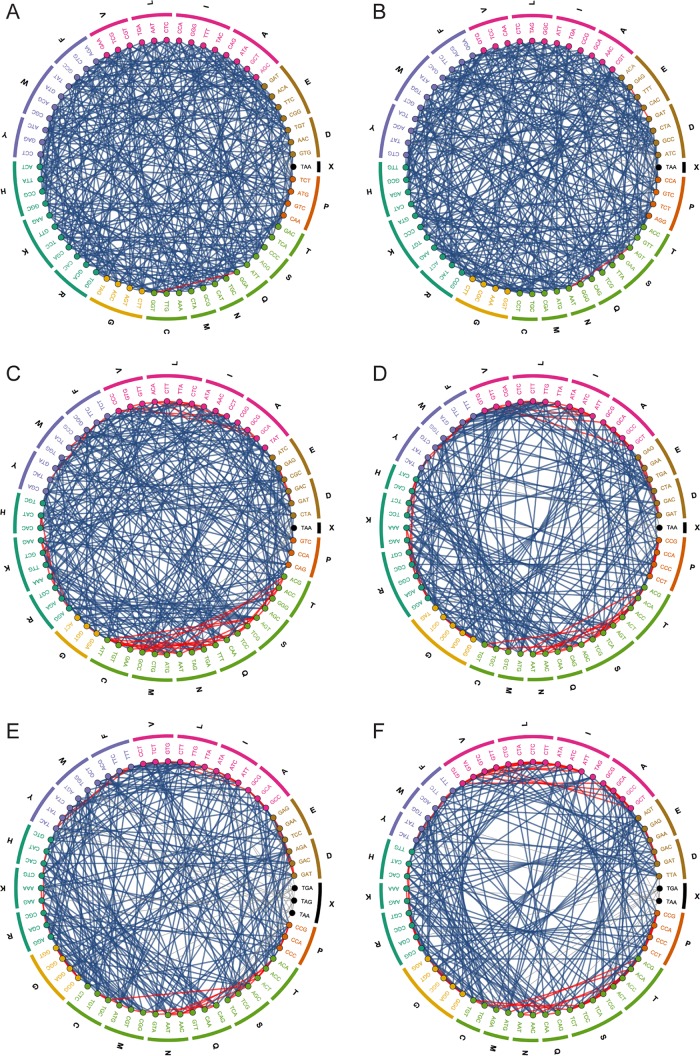
Accessibility plots of the genetic codes presented in this study. Plots are organized as described for Fig. 1B. (A) The optimal code (OPT). (B) The optimal code with the imposed native codon restriction (OPR-NR). (C) Change-minimizing code utilizing a linear penalty for changes (CMC). (D) Change-minimizing code utilizing a power of 2 penalty for changes (CMC^2^). (E) Genetic code derived from a recursive approach of amino acid reassignment (REC). (F) the resulting genetic code of reassigning the seven free codons, as described by Ostrov et al. (50).

Applying the selection procedure outlined here (for further details, see Materials and Methods) yields genetic codes with a significantly higher level of codon accessibility concerning both the number (Fig. S1A) and the chemical diversity of the SNR-obtained amino acids (optimized [OPT] code (Fig. 2A), note the decrease in red edges from 109 to 1). The recoded genetic code includes a single stop codon manually added after the selection procedure. Increased amino acid accessibility naturally leads to a concomitant decrease in robustness to mutation by allowing SNRs to lead to a wider variety of amino acid substitutions (Fig. S1A), given the inverse relationship of these properties. The bias in codon distribution in the standard code between different amino acids had been flattened such that the number of codons assigned for amino acids was between 2 and 4, compared to assignments from 1 to 6 in the standard code. Adding a native codon restriction to ensure that each amino acid has at least one native codon produces the OPT-NR code, which is slightly less optimized but still has a higher degree of fitness than the standard code (as defined by the genetic algorithm [Fig. 2B; Fig. S1B]). The distribution of the inter- and intra-chemical class SNR conversions is shown in Fig. S2.

10.1128/mBio.01654-17.1 FIG S1  Tables representing the different genetic codes described in this study. Colors are indicative of the amino chemical classes, and numbers in parentheses indicate the number of SNR-accessible unique amino acids for each codon (similar to that in Fig. 1A). (A) The optimal code. (B) The optimal code with the imposed native codon restriction. (C) Change-minimizing code utilizing a linear penalty for changes. (D) Change-minimizing code utilizing a power of 2 penalty for changes. (E) Genetic code derived from a recursive approach of amino acid reassignment. (F) The resulting genetic code of reassigning the seven free codons, as described by Ostrov et al. (50). Download FIG S1, EPS file, 1.4 MB. Copyright © 2017 Pines et al. 2017 Pines et al. This content is distributed under the terms of the Creative Commons Attribution 4.0 International license.

10.1128/mBio.01654-17.2 FIG S2  Edge-weight distributions of SNR-accessible amino acids. Edge counts are shown for every genetic code discussed in this study. Colors are as for Fig. 1B and correspond to whether the amino acid change is within the same chemical class (red) or between classes (blue). Edges connecting to stop codons are shaded in gray. Download FIG S2, EPS file, 0.9 MB. Copyright © 2017 Pines et al. 2017 Pines et al. This content is distributed under the terms of the Creative Commons Attribution 4.0 International license.

While these genetic codes represent maximal accessibility via SNRs, they require 60 and 43 codon reassignments for the OPT and OPT-NR values, respectively. Genetic code refactoring on such a scale is not likely to be feasible in the near future, due to the complexities of tRNA reassignments, simultaneously recoding genes while preserving key properties, such as secondary structure, ribosome binding sites, and other features (see Discussion). A second fitness function that penalizes codon reassignments in the effort to balance maximal accessibility with a minimal number of reassignments resulted in another two genetic codes termed CMC (change-minimizing code) and CMC^2^. These codes, require 30 and 9 reassignments via linear and power penalties but with 25.1% and 10.2% improved SNR accessibility (F_unique_) relative to the standard code nonetheless (Fig. 2C and D). Genetic code tables and the chemical differences for SNR-accessible amino acids are shown in Fig. S1C and D and S2. Given that minimal changes substantially improve each component of our fitness function (Fig. S3), it may be possible to create a platform organism with a small number of reassigned codons that achieves the lion’s share of possible accessibility improvement.

10.1128/mBio.01654-17.3 FIG S3  Genetic codes fitness. (A) The fitness increase is plotted versus the number of rounds made by the genetic algorithm (for OPT, OPT-NR, CMC, CMC^2^, and the rE. coli-57 codes) or the recursive approach (REC). Note that the recursive approach maximizes its optimization after 16 rounds, as indicated in the lower plot. (B) The fitness values of the chemical diversity components of the fitness function of each genetic code were divided by the number of required codon reassignments and plotted against the number of reassignments. The diagram illustrates the rapid decrease in marginal value per reassignment Download FIG S3, EPS file, 2.2 MB. Copyright © 2017 Pines et al. 2017 Pines et al. This content is distributed under the terms of the Creative Commons Attribution 4.0 International license.

A second approach for increased evolvability involves modifying the genetic code, one that might prove more practical, is a stepwise reassignment of amino acids. This process entails the identification of the optimal amino acid replacement(s) and then reevaluating the resulting altered code until no further single-step improvement is possible. Since there are often many amino acid reassignments that yield equivalent fitness improvements, we implemented a recursive branching approach to exhaustively evaluate all potential single-step reassignment procedures. Using this method, the code’s fitness reached its maximum after 16 recursive rounds and resulted in 14.8% improvement in unique amino acid accessibility compared to the standard code (Fig. 2E; Fig. S1E and S3A). This code exhausted all available single-step improvements, demonstrating that multiple simultaneous mutations are necessary to improve the code further. While this approach resulted in less-evolvable codes than the OPT codes (see below), this approach may be more practical for experimental evaluations in the near future as it utilizes iterative codon reassignment rather than simultaneous wholesale code engineering.

### Maximizing accessibility for existing recoded organisms.

Genome-wide multiple reassignments are far from trivial and may result in a nonviable organism that is challenging to test and modify. There are currently only a few organisms with artificially modified genetic codes and the attendant genome refactoring, one of which may be ideal to test our approach for increasing SNR accessibility. Ostrov et al. recently reported on the ongoing effort to engineer a 57-codon *E. coli* genome (rE. coli-57) (50), with the seven codons replaced with their synonymous counterparts. We applied our genetic algorithm to reassign these seven codons for fitness maximization (Fig. 2F; Fig. S1F) and generated a code with a 7.7% increase in unique amino acid accessibility and 9.6% increase in chemical class diversity obtainable by SNRs. While these differences are relatively small, they represent a significant increase in the complexity accessible by random mutation and allow for new paths on the fitness landscape of interest to be explored. The tRNA reassignments required for the proposed code should not affect the physiology of the final rE. Coli-57 strain, since these codons are deleted from this genome. Following successful reassignment, codons may be gradually reintroduced to the genome by employing MAGE or other methods (49, 53, 54). This process and the attendant debugging required will enable better understanding of the reassignment design rules, and this may finally lead to the synthesis of a rationally redesigned genome by employing a novel genetic code.

### Genetic code analysis.

**FIG 3:**
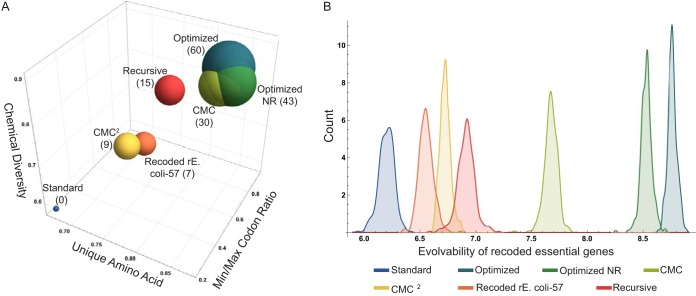
Comparisons of the genetic codes presented in this study. (A) A 3-dimensional bubble chart with the axes corresponding to the three parameters that were selected for optimization. Bubble size corresponds to the number of codon-amino acid reassignments required for each genetic code, which is also indicated in parentheses. (B) Genome evolvability of the presented codes, computed based on the accessibility of the recoded collection of the *E. coli* essential genes.

The genetic codes described here can be compared both at the level of code fitness and with the broader term of genome evolvability. Figure 3A places all the genetic codes outlined in this study in a 3-dimensional space composed of the three parameters that were subject to optimization, namely, number of unique SNR-accessible amino acids, their chemical diversity, and the distribution of the number of codons assigned for an amino acid. Every genetic code is represented as a bubble, with its size proportional to the number of the required reassignments. The OPT, OPT-NR, and CMC codes partially overlap and cluster close to the most optimized corner of the space. The recursive code is less optimized but is still improved relative to the CMC code, which utilizes a power penalty for reassignments. The recoded rE. coli-57 code represents a different combination of optimizations than its neighbor, the CMC^2^ code, and requires the lowest number of reassignments. Two-dimensional versions of Fig. 3A are depicted in Fig. S4A to C.

10.1128/mBio.01654-17.4 FIG S4  A set of 2-dimensional bubble charts comparing the three different fitness components comprising the fitness function described in this study. As in Fig. 3, bubble size is proportional to the number of required reassignments Download FIG S4, EPS file, 2.3 MB. Copyright © 2017 Pines et al. 2017 Pines et al. This content is distributed under the terms of the Creative Commons Attribution 4.0 International license.

As the genetic code and genome content are inextricably intertwined in living organisms, the distribution of codons observed in living organisms is the result of continual selection over billions of years for genetic stability, efficient transcription, translation, and regulation. Recoding genetic material using these artificial codes, therefore, must preserve inherent structural and sequence features found in the genome as much as possible. To evaluate the genome-wide effects of recoding, we implemented a resequencing algorithm that seeks to minimize predicted changes in folding energy and secondary structure (see Materials and Methods) and then recoded the set of known essential *E. coli* genes by using a range of generated genetic codes for analysis. We therefore defined an “evolvability” score calculated according to equation 5, which iterates over the length of a protein and sums the number of unique amino acids that are accessible from a given codon i (*D* = 1, required for an SNR to convert C to C′) and normalizes the result by the length of the protein P.

(5) $$E=1/\|\text{P}\|\Sigma \delta_{\text{ij}}*\text{num }(\text{AA}(C_{j}))=1\;\text{if D}({\text{C}}_{i},{\text{C}}_{j})==1\text{ else }0$$

Wild-type genes were found to have lower, more variable evolvability scores than the genes recoded using the generated codes, in rough concordance to their calculated fitness scores (Fig. 3B; Fig. S3A); higher fitness codes permit greater sequence flexibility than the more constrained standard code. However, since synonymous codon recoding is now feasible on a large scale (49, 53, 55), it may be possible to selectively recode portions of the genome such that they use these reassigned codons more frequently.

## DISCUSSION

The modern genetic code buffer errors both in terms of mutations and translation, and as a result it limits SNR-based amino acid accessibility (Fig. 1A and B) (12–14, 56). This inherent property of the genetic code significantly limits the accessibility of error-based methods, preventing the exploration of many adaptive mutations, including at times the discovery of the most favorable mutant (Fig. 1C and D). Here, we have described more flexible genetic codes with access to larger portions of the full fitness landscape, with the aim of generating a more evolvable organism, primarily for adaptive laboratory evolution experiments. While the codes discovered by our genetic algorithm presented a high degree of accessibility, they required multiple amino acid reassignments (Fig. 2 and 3; Fig. S1). Using a second approach, we adopted a stepwise strategy, with each step reassigning an amino acid-codon pair in a manner that increased accessibility the most. This resulted in a less-accessible final genetic code than the optimized ones discovered using the genetic algorithm, but it had the advantage of gradual modifications and might prove to be more practical experimentally (Fig. 2E and 3; Fig. S1E). Finally, we reassigned the seven free codons of the recently reported rE.coli-57 strain (50), which increased unique amino acid accessibility by 7.7% (Fig. 2F and 3; Fig. S1F) and is currently the most practical approach to test the hypothesis described here. Altogether, the genetic codes presented here demonstrate a significantly higher level of fitness regarding the number of unique SNR-accessible amino acids, their chemical properties, and the codon assignment distribution (Fig. 3A). This fitness increase may be further translated to the consequent increase in the overall evolvability at the genome level when essential genes are recoded according to these genetic codes (Fig. 3B). In general, the marginal value of codon reassignments, especially for improvements in chemical class accessibility, decreases rapidly when using the genetic algorithm (Fig. S3B), suggesting that the optimal effort-reward tradeoff must be determined to the extent of reengineering that can be tolerated.

High-throughput saturation mutagenesis methods already provide access to all possible 19 amino acids and are applicable for the introduction of many single point mutations across large DNA segments. Indeed, application of such methods, while still new, has already identified novel relevant mutations and insights, overcoming the amino acid accessibility problem we described here (31–33). These approaches, combined with error-based methods such as error-prone PCR, can efficiently explore multiple mutations across a single gene or a few genes without the laborious refactoring of the genetic code. Hence, we predict that refactored organisms, such as the ones described here, will contribute the most when submitted to adaptive laboratory evolution experiments, in which multiple mutations are expected to occur across the whole genome.

The physical engineering of a wholly recoded organism must be approached from two directions: engineering of the actual translation apparatus (tRNAs, aminoacyl-tRNA synthetases [aaRS], release factors, etc.) and recoding of the physical genome to utilize the reassigned codons to maintain a viable proteome. The range of existent alternative genetic codes (57) shows that at least some degree of recoding is biochemically feasible and occurs naturally, albeit its focus on reassigning serine, arginine, and stop codons. Recoding on the scale proposed here would likely require breaking the degeneracy of the genetic code such that there was a one-to-one mapping of tRNAs to their cognate codons, which could then be reassigned as desired (58). Since all sequenced organisms to date rely on wobble interactions or chemical base modification to allow individual tRNAs to decode multiple codons (59–61), the switch from a translation system relying on inexact sequence recognition to one where the tRNA anticodon is associated with only a single possible codon will pose daunting engineering challenges. These include the identification or directed evolution of aaRS enzymes that only recognize a cognate, unmodified tRNA, coupled with host engineering to remove or change the specificity of base-modifying enzymes, such that the organism remains viable with the expression of the modified tRNA-aaRS pairs. However, both engineering and directed evolution of arbitrary tRNA-aaRS-amino acid pairings is already common for nonstandard amino acids (62–66), and systematic engineering of tRNAs to avoid base modification may bear fruit.

Beyond refactoring of the translation system, the genome itself must be synthesized and recoded so that the proteome remains identical, sequence-wise. The cost of DNA synthesis is continuously decreasing, enabling large-scale assemblies to be executed in laboratory environments (67, 68). Whole-genome chemical synthesis was previously shown with *Mycoplasma mycoides* (69), and recently a smaller version of this genome was shown to be viable after removing many of its nonessential genes (70). Moreover, genome synthesis has been successfully demonstrated in a variety of organisms spanning from synthetic viral genomes to yeast chromosomes and mouse mitochondria (71–73). Notably, leading members of the synthetic biology community have recently proposed synthesis of the complete human genome, following the “learn through building” paradigm (74). Still, even the successful synthesis of the first step in our recursive approach is currently challenging.

The viability of an entirely recoded organism is not ensured; the challenge of bootstrapping an altered code and the required debugging of the resulting deleterious or lethal mutations remain unsolved challenges. Unlike the rE. coli-57 strain, which was constructed in several steps, allowing identification of detrimental designs, codon reassignments should be made simultaneously across the whole genome. We still are not familiar with the complete functionalities of the full genomic sequence of an organism, and a complete recoding may result in severely impaired fitness. Though it might be feasible that once a recoded organism is constructed, adaptive evolution can be used to select for genome remodeling that improves strain fitness closer to wild-type levels (75). Moreover, working with the rE. coli-57 strain, gradually reintroducing codons with reassigned amino acids may help in overcoming this challenge. Recoding a whole genome with amino acid reassignments will prove to be significantly more complicated than previously reported synonymous reassignments (49, 50). Moreover, the genetic codes proposed here eliminate the robustness of the standard code and disrupt the fine balance between robustness and evolvability (76, 77). This may lead to a disruption of the genomic integrity that decreases host viability. However, given that the estimates of *E. coli*’s point mutation rate are generally on the order of 10^−10^/bp/generation (78), this effect will be more obvious under stress-induced mutagenesis or with exposure to DNA mutagens than with replication on laboratory time scales. Finally, protein mistranslation may pose an additional concern. While the genetic code structure buffers some translational errors, studies have shown that mistranslations are well tolerated, highly regulated, and also beneficial at times (79–83). It remains to be determined whether recoded organisms will be able to tolerate increased error rates in translation.

Any organisms with an artificial coding scheme for DNA-protein translation have the implicit benefit of strong biocontrol. Accidental releases of an organism altered in this fashion cannot easily express environmental DNA and cannot transfer heterologous DNA to other microbes, due to the drastic differences in their coding schemes. On the other hand, considering its potential resistance to viral infections (84), a release of such an organism to the environment is a concern. Suitable safety measures, such as dependence on exogenously supplied ligands, suicide circuits, or nuclease-based DNA destruction, should be implemented (85, 86). Another approach is to induce addiction to an environmentally unavailable nonstandard amino acid, ensuring that its escape from the laboratory environment would result in cell death (87, 88). This seems particularly fitting to the case presented here, since it requires genome recoding and freeing of at least a single codon for reassignment purposes, which could be easily accommodated into such designs.

### Conclusions.

We have presented here the concept of refactoring the genetic code with the aim of increasing evolvability by optimizing the SNR-accessible amino acids in terms of both the number of unique amino acids and their chemical properties. We added a third parameter to our fitness function to avoid codon bias (Fig. 3A). It is possible to alter these settings, such as by using different chemical property classes or adding more dimensions, such as amino acid size, secondary structure propensity, or molecular weight, etc. Other options include directing the genetic code toward a different bias than the one that exists in the standard code or making a less evolvable genetic code for maintaining desirable traits.

A successful synthesis of such an organism may result in extreme evolvability with increased accessibility to a significantly larger portion of the fitness landscape, enhancing the effect of SNR-dependent directed evolution methods for protein engineering and strain design purposes. Once the desired phenotype is isolated, the corresponding amino acid sequence can be reverse-engineered to the standard code for incorporation in natural, more stable organisms. In addition, such an organism may serve as a platform for studying more fundamental questions, such as the evolvability of the genetic code and evolutionary robustness.

## MATERIALS AND METHODS

### Computational details.

All simulations were run on a T430 Thinkpad (Windows 8.1; 16 GB RAM) with Python 2.7.11. The genetic algorithm described below was run using the PyPy 5.4.1 interpreter, while all other scripts were run using the standard Python 2.7.11 interpreter. RNAFold (89) was used to compute secondary structures and folding energies for all sequences. Visualizations were generated using Mathematica 10.

### Calculation of codon accessibility.

The average number of SNR-accessible amino acid transitions was calculated by computing the number of codons encoding different unique amino acids with a Hamming distance of 1 per amino acid and then averaging the result. For the standard *E. coli* genetic code, each codon can be converted into 6.1 unique amino acids by SNRs, on average.

### Genetic algorithm for code generation.

The genetic algorithm was used to evolve the native genetic code (referred to as the standard genetic code in our text) into codes that maximize the number of alternative amino acids with distinct chemical classes accessible by mutating codon C with only SNRs. The full implementation of the genetic algorithm is provided in the repository reported in the “Data availability” section, below; only a general description is included here.

The fitness function used for the genetic algorithm has three components. First, the number of unique amino acids accessible by SNRs averaged over all codons in the genetic code, F_unique_, is used to select for codes where SNRs lead to the greatest diversity of amino acids that can be obtained by mutating a single codon. Next, the number of mutations leading to the least accessible amino acid divided by the number of mutations leading to the most accessible amino acid, the F_ratio_, is included as part of the fitness function to prevent situations where nearly all codons are assigned to a single amino acid; codes with higher values of F_ratio_ will have more even distributions of codons between amino acids. Finally, we included a chemical diversity score, F_chem_, that counts the number of nondistinct amino acid chemical classes accessible from a given codon by SNRs. The overall fitness function is the product of these individual components, F = F_unique_ F_ratio_ F_chem_ divided by their theoretical maximum F_unique, max_ = 9, F_ratio_ = 1.0, F_chem_ = 9 to avoid inappropriate weighting during selection. Amino acid chemical class definitions were obtained from the amino acid reference chart of Sigma. Codon reassignments can also be penalized by reducing F via a factor alpha^N, where N = 1 for linear penalties and N = 2 for square penalties, resulting in the CMC and CMC^2^, respectively.

Each simulation utilized 2,500 individuals and lasted 1,000 rounds. For codes that excluded the stop codon from the genetic algorithm procedure, TAG and TGA were arbitrarily reassigned to asparagine and TAA was reserved as the sole stop codon available. The codes for the next round of selection were generated using random mutation of the top 10% in a cyclical manner. The previous winner was also retained in the population undergoing selection to avoid fitness regression during the procedure. Since our goal was to select codes with optimized chemical diversity, an additional check was added to accept only new codes that had at least the same level of diversity (F_chem_) as the previous best observed in the simulation. Codes can be subject to a range of constraints, including fixed amino acids, requiring at least one codon to retain the wild-type amino acid assignment, and permitting the removal of amino acids during the selection process. The utilization of these flags is specified in the code. During all simulations, the mutation rate was 10% per codon (i.e., 10% chance that a codon would have its amino acid assignment swapped with another randomly chosen codon). Our empirical results indicated that this mutation rate generally yielded rapid improvements without trapping codes within local fitness minima due to extreme mutation rates.

### Single-step code generation.

As an alternative to the genetic algorithm, we implemented a “best-move-based” improvement scheme that iteratively improved the standard genetic code. Briefly, all possible codon reassignments for an input code were considered. To exhaustively evaluate possible best-move codes, we implemented a recursive branching algorithm that generated all possible single move codes with equivalent fitness improvements until no further accessibility improvement was possible; the final ensemble of codes was then analyzed to find the set of those with maximized fitness. Stop codons were not included as part of the search but were added back after all moves had been exhausted.

### Gene resequencing with generated codes.

Once candidate codes were generated using the genetic algorithm or recursive approach outlined above, we next recoded the set of known *E. coli* essential genes to use these new codon-amino acid mappings. Given previous data showing that the secondary structure of the 4 bp of the 5′ untranslated region along with the first 37 bp of a coding mRNA had the largest impact on translation efficiency (90), we split the recoding process into two parts. First, the coding section of the mRNA (padded out to 39 bp) was translated according to the standard code into the corresponding polypeptide sequence; noncoding bases were not altered. The translated sequences were then converted back into a sequence of candidate codons by using the generated genetic codes, yielding a sequence of lists. Using the python itertools library, we built the Cartesian product of these possible codon combinations for the truncated mRNA and scanned 1% of all possible assembled sequences to identify the one that best matched the wild-type folding energy and secondary structure. Ideally, this selection scheme would theoretically maintain the same transcription and translation efficiencies observed for the wild-type sequence, but it does neglect, for the sake of simplicity, the preservation of other sequence features (ribosome binding sites, for example). Once this initial scan is complete, areas where the recoded mRNA leader and the original mRNA sequences differ in secondary structure are identified, and alternative codons from the new proposed code are substituted to reduce the structural disparity.

After the recoded leader sequence is finalized, the entire candidate gene is then translated from the original standard code to the new candidate coding scheme. The process of whole-gene recoding is conceptually similar to that outlined for the leader sequence, but with several key differences due to the larger scale of the problem. It is not feasible to compute or scan the Cartesian product of all possible codon combinations for a coding sequence due to a combinatorial explosion of candidates, so instead of analyzing the whole sequence at once, a sliding window approach is used to reduce the number of candidate sequences. The leader sequence of each essential gene is replaced with its recoded counterpart (computed as described above), followed by sequence optimization of 30-bp slices at once by using the same approach employed for the mRNA leader sequences. Only 0.4% of all possible candidates are screened, to reduce the computational burden of sequence exploration, although it is feasible to increase the number of candidates examined depending on the availability of computational resources. Once all windows within the sequence were analyzed, there was no further adjustment to remove discrepancies between the wild-type and recoded sequence structural properties. Other more comprehensive methods are also available (50) that may produce improved recoded gene candidates.

### Data availability.

Code and data used in this study are deposited in the https://bitbucket.org/jdwinkler/genetic_code_generator/ repository for download.

## References

[1] Nirenberg M, Caskey T, Marshall R, Brimacombe R, Kellogg D, Doctor B, Hatfield D, Levin J, Rottman F, Pestka S, Wilcox M, Anderson F 1966 The RNA code and protein synthesis. Cold Spring Harb Symp Quant Biol 31:11–24. doi:10.1101/SQB.1966.031.01.008. 5237186

[2] Woese CR 1965 Order in the genetic code. Proc Natl Acad Sci U S A 54:71–75. doi:10.1073/pnas.54.1.71. 5216368 PMC285798

[3] Woese CR, Dugre DH, Dugre SA, Kondo M, Saxinger WC 1966 On the fundamental nature and evolution of the genetic code. Cold Spring Harb Symp Quant Biol 31:723–736. doi:10.1101/SQB.1966.031.01.093. 5237212

[4] Alff-Steinberger C 1969 The genetic code and error transmission. Proc Natl Acad Sci U S A 64:584–591. doi:10.1073/pnas.64.2.584. 5261915 PMC223384

[5] Haig D, Hurst LD 1991 A quantitative measure of error minimization in the genetic code. J Mol Evol 33:412–417. doi:10.1007/BF02103132. 1960738

[6] Crick FH 1968 The origin of the genetic code. J Mol Biol 38:367–379. doi:10.1016/0022-2836(68)90392-6. 4887876

[7] Wong JT 1975 A co-evolution theory of the genetic code. Proc Natl Acad Sci U S A 72:1909–1912. doi:10.1073/pnas.72.5.1909. 1057181 PMC432657

[8] Di Giulio M 2016 An autotrophic origin for the coded amino acids is concordant with the coevolution theory of the genetic code. J Mol Evol 83:93–96. doi:10.1007/s00239-016-9760-x. 27743002

[9] Taylor FJ, Coates D 1989 The code within the codons. Biosystems 22:177–187. doi:10.1016/0303-2647(89)90059-2. 2650752

[10] Freeland SJ, Hurst LD 1998 The genetic code is one in a million. J Mol Evol 47:238–248. doi:10.1007/PL00006381. 9732450

[11] Knight RD, Freeland SJ, Landweber LF 1999 Selection, history and chemistry: the three faces of the genetic code. Trends Biochem Sci 24:241–247. doi:10.1016/S0968-0004(99)01392-4. 10366854

[12] Freeland SJ, Knight RD, Landweber LF, Hurst LD 2000 Early fixation of an optimal genetic code. Mol Biol Evol 17:511–518. doi:10.1093/oxfordjournals.molbev.a026331. 10742043

[13] Sella G, Ardell DH 2002 The impact of message mutation on the fitness of a genetic code. J Mol Evol 54:638–651. doi:10.1007/s00239-001-0060-7. 11965436

[14] Freeland SJ, Wu T, Keulmann N 2003 The case for an error minimizing standard genetic code. Orig Life Evol Biosph 33:457–477. doi:10.1023/A:1025771327614. 14604186

[15] Myers RM, Lerman LS, Maniatis T 1985 A general method for saturation mutagenesis of cloned DNA fragments. Science 229:242–247. doi:10.1126/science.2990046. 2990046

[16] Miyazaki K, Arnold FH 1999 Exploring nonnatural evolutionary pathways by saturation mutagenesis: rapid improvement of protein function. J Mol Evol 49:716–720. doi:10.1007/PL00006593. 10594172

[17] Pines G, Pines A, Garst AD, Zeitoun RI, Lynch SA, Gill RT 2015 Codon compression algorithms for saturation mutagenesis. ACS Synth Biol 4:604–614. doi:10.1021/sb500282v. 25303315

[18] Hu S, Huang J, Mei L, Yu Q, Yao S, Jin Z 2010 Altering the regioselectivity of cytochrome P450 BM-3 by saturation mutagenesis for the biosynthesis of indirubin. J Mol Catal B Enzym 67:29–35. doi:10.1016/j.molcatb.2010.07.001.

[19] Rui L, Reardon KF, Wood TK 2005 Protein engineering of toluene ortho-monooxygenase of Burkholderia cepacia G4 for regiospecific hydroxylation of indole to form various indigoid compounds. Appl Microbiol Biotechnol 66:422–429. doi:10.1007/s00253-004-1698-z. 15290130

[20] Fan A, Li SM 2016 Saturation mutagenesis on Arg244 of the tryptophan C4-prenyltransferase FgaPT2 leads to enhanced catalytic ability and different preferences for tryptophan-containing cyclic dipeptides. Appl Microbiol Biotechnol 100:5389–5399. doi:10.1007/s00253-016-7365-3. 26875876

[21] Xu X, Chen J, Wang Q, Duan C, Li Y, Wang R, Yang S 2016 Mutagenesis of key residues in the binding center of l-aspartate-β-semialdehyde dehydrogenase from Escherichia coli enhances utilization of the cofactor NAD(H). Chembiochem 17:56–64. doi:10.1002/cbic.201500534. 26662025

[22] Qiu YL, He QH, Xu Y, Wang W, Liu YY 2016 Modification of a deoxynivalenol-antigen-mimicking nanobody to improve immunoassay sensitivity by site-saturation mutagenesis. Anal Bioanal Chem 408:895–903. doi:10.1007/s00216-015-9181-5. 26608283

[23] Cahn JKB, Baumschlager A, Brinkmann-Chen S, Arnold FH 2016 Mutations in adenine-binding pockets enhance catalytic properties of NAD(P)H-dependent enzymes. Protein Eng Des Sel 29:31–38. doi:10.1093/protein/gzv057. 26512129 PMC4678007

[24] Asad S, Dastgheib SMM, Khajeh K 2016 Construction of a horseradish peroxidase resistant toward hydrogen peroxide by saturation mutagenesis. Biotechnol Appl Biochem 63:789–794. doi:10.1002/bab.1437. 26331237

[25] White PJ, Squirrell DJ, Arnaud P, Lowe CR, Murray JA 1996 Improved thermostability of the North American firefly luciferase: saturation mutagenesis at position 354. Biochem J 319:343–350. doi:10.1042/bj3190343. 8912666 PMC1217775

[26] Cormack BP, Valdivia RH, Falkow S 1996 FACS-optimized mutants of the green fluorescent protein (GFP). Gene 173:33–38. doi:10.1016/0378-1119(95)00685-0. 8707053

[27] Firnberg E, Labonte JW, Gray JJ, Ostermeier M 2014 A comprehensive, high-resolution map of a gene’s fitness landscape. Mol Biol Evol 31:1581–1592. doi:10.1093/molbev/msu081. 24567513 PMC4032126

[28] Kitzman JO, Starita LM, Lo RS, Fields S, Shendure J 2015 Massively parallel single-amino-acid mutagenesis. Nat Methods 12:203–206. doi:10.1038/nmeth.3223. 25559584 PMC4344410

[29] Stiffler MA, Hekstra DR, Ranganathan R 2015 Evolvability as a function of purifying selection in TEM-1 β-lactamase. Cell 160:882–892. doi:10.1016/j.cell.2015.01.035. 25723163

[30] Wrenbeck EE, Klesmith JR, Stapleton JA, Adeniran A, Tyo KEJ, Whitehead TA 2016 Plasmid-based one-pot saturation mutagenesis. Nat Methods 13:928–930. doi:10.1038/nmeth.4029. 27723752 PMC5666567

[31] Whitehead TA, Chevalier A, Song Y, Dreyfus C, Fleishman SJ, De Mattos C, Myers CA, Kamisetty H, Blair P, Wilson IA, Baker D 2012 Optimization of affinity, specificity and function of designed influenza inhibitors using deep sequencing. Nat Biotechnol 30:543–548. doi:10.1038/nbt.2214. 22634563 PMC3638900

[32] Garst AD, Bassalo MC, Pines G, Lynch SA, Halweg-Edwards AL, Liu R, Liang L, Wang Z, Zeitoun R, Alexander WG, Gill RT 2017 Genome-wide mapping of mutations at single-nucleotide resolution for protein, metabolic and genome engineering. Nat Biotechnol 35:48–55. doi:10.1038/nbt.3718. 27941803

[33] Firnberg E, Ostermeier M 2013 The genetic code constrains yet facilitates Darwinian evolution. Nucleic Acids Res 41:7420–7428. doi:10.1093/nar/gkt536. 23754851 PMC3753648

[34] Reetz MT, Carballeira JD 2007 Iterative saturation mutagenesis (ISM) for rapid directed evolution of functional enzymes. Nat Protoc 2:891–903. doi:10.1038/nprot.2007.72. 17446890

[35] Reetz MT, Kahakeaw D, Lohmer R 2008 Addressing the numbers problem in directed evolution. Chembiochem 9:1797–1804. doi:10.1002/cbic.200800298. 18567049

[36] Romero PA, Arnold FH 2009 Exploring protein fitness landscapes by directed evolution. Nat Rev Mol Cell Biol 10:866–876. doi:10.1038/nrm2805. 19935669 PMC2997618

[37] Voigt CA, Mayo SL, Arnold FH, Wang ZG 2001 Computational method to reduce the search space for directed protein evolution. Proc Natl Acad Sci U S A 98:3778–3783. doi:10.1073/pnas.051614498. 11274394 PMC31129

[38] Kumar P, Henikoff S, Ng PC 2009 Predicting the effects of coding non-synonymous variants on protein function using the SIFT algorithm. Nat Protoc 4:1073–1081. doi:10.1038/nprot.2009.86. 19561590

[39] Hecht M, Bromberg Y, Rost B 2015 Better prediction of functional effects for sequence variants. BMC Genomics 16:S1. doi:10.1186/1471-2164-16-S8-S1. PMC4480835 26110438

[40] Toprak E, Veres A, Michel JB, Chait R, Hartl DL, Kishony R 2011 Evolutionary paths to antibiotic resistance under dynamically sustained drug selection. Nat Genet 44:101–105. doi:10.1038/ng.1034. 22179135 PMC3534735

[41] Tenaillon O, Rodríguez-Verdugo A, Gaut RL, McDonald P, Bennett AF, Long AD, Gaut BS 2012 The molecular diversity of adaptive convergence. Science 335:457–461. doi:10.1126/science.1212986. 22282810

[42] Barrick JE, Lenski RE 2013 Genome dynamics during experimental evolution. Nat Rev Genet 14:827–839. doi:10.1038/nrg3564. 24166031 PMC4239992

[43] Winkler JD, Garcia C, Olson M, Callaway E, Kao KC 2014 Evolved osmotolerant Escherichia coli mutants frequently exhibit defective N-acetylglucosamine catabolism and point mutations in cell shape-regulating protein MreB. Appl Environ Microbiol 80:3729–3740. doi:10.1128/AEM.00499-14. 24727267 PMC4054140

[44] Winkler JD, Halweg-Edwards AL, Erickson KE, Choudhury A, Pines G, Gill RT 2016 The resistome: a comprehensive database of Escherichia coli resistance phenotypes. ACS Synth Biol 5:1566–1577. doi:10.1021/acssynbio.6b00150. 27438180

[45] Wagner J, Gruz P, Kim SR, Yamada M, Matsui K, Fuchs RP, Nohmi T 1999 The dinB gene encodes a novel E. coli DNA polymerase, DNA pol IV, involved in mutagenesis. Mol Cell 4:281–286. doi:10.1016/S1097-2765(00)80376-7. 10488344

[46] Tang M, Pham P, Shen X, Taylor JS, O’Donnell M, Woodgate R, Goodman MF 2000 Roles of E. coli DNA polymerases IV and V in lesion-targeted and untargeted SOS mutagenesis. Nature 404:1014–1018. doi:10.1038/35010020. 10801133

[47] Rosenberg SM 2001 Evolving responsively: adaptive mutation. Nat Rev Genet 2:504–515. doi:10.1038/35080556. 11433357

[48] Watson M, Liu JW, Ollis D 2007 Directed evolution of trimethoprim resistance in Escherichia coli. FEBS J 274:2661–2671. doi:10.1111/j.1742-4658.2007.05801.x. 17451440

[49] Isaacs FJ, Carr PA, Wang HH, Lajoie MJ, Sterling B, Kraal L, Tolonen AC, Gianoulis TA, Goodman DB, Reppas NB, Emig CJ, Bang D, Hwang SJ, Jewett MC, Jacobson JM, Church GM 2011 Precise manipulation of chromosomes in vivo enables genome-wide codon replacement. Science 333:348–353. doi:10.1126/science.1205822. 21764749 PMC5472332

[50] Ostrov N, Landon M, Guell M, Kuznetsov G, Teramoto J, Cervantes N, Zhou M, Singh K, Napolitano MG, Moosburner M, Shrock E, Pruitt BW, Conway N, Goodman DB, Gardner CL, Tyree G, Gonzales A, Wanner BL, Norville JE, Lajoie MJ, Church GM 2016 Design, synthesis, and testing toward a 57-codon genome. Science 353:819–822. doi:10.1126/science.aaf3639. 27540174

[51] Gilis D, Massar S, Cerf NJ, Rooman M 2001 Optimality of the genetic code with respect to protein stability and amino-acid frequencies. Genome Biol 2:research0049. doi:10.1186/gb-2001-2-11-research0049. PMC60310 11737948

[52] Santos J, Monteagudo A 2011 Simulated evolution applied to study the genetic code optimality using a model of codon reassignments. BMC Bioinformatics 12:56. doi:10.1186/1471-2105-12-56. 21338505 PMC3053255

[53] Wang HH, Isaacs FJ, Carr PA, Sun ZZ, Xu G, Forest CR, Church GM 2009 Programming cells by multiplex genome engineering and accelerated evolution. Nature 460:894–898. doi:10.1038/nature08187. 19633652 PMC4590770

[54] Bassalo MC, Garst AD, Halweg-Edwards AL, Grau WC, Domaille DW, Mutalik VK, Arkin AP, Gill RT 2016 Rapid and efficient one-step metabolic pathway integration in E. coli. ACS. ACS Synth Biol 5:561–568. doi:10.1021/acssynbio.5b00187. 27072506

[55] Wang K, Fredens J, Brunner SF, Kim SH, Chia T, Chin JW 2016 Defining synonymous codon compression schemes by genome recoding. Nature 539:59–64. doi:10.1038/nature20124. 27776354 PMC5321499

[56] Woese CR 1965 On the evolution of the genetic code. Proc Natl Acad Sci U S A 54:1546–1552. doi:10.1073/pnas.54.6.1546. 5218910 PMC300511

[57] Knight RD, Freeland SJ, Landweber LF 2001 Rewiring the keyboard: evolvability of the genetic code. Nat Rev Genet 2:49–58. doi:10.1038/35047500. 11253070

[58] Kwon I, Kirshenbaum K, Tirrell DA 2003 Breaking the degeneracy of the genetic code. J Am Chem Soc 125:7512–7513. doi:10.1021/ja0350076. 12812480

[59] Crick FHC 1966 Codon-anticodon pairing: the wobble hypothesis. J Mol Biol 19:548–555. doi:10.1016/S0022-2836(66)80022-0. 5969078

[60] Dunin-Horkawicz S, Czerwoniec A, Gajda MJ, Feder M, Grosjean H, Bujnicki JM 2006 MODOMICS: a database of RNA modification pathways. Nucleic Acids Res 34:D145–D149. doi:10.1093/nar/gkj084. 16381833 PMC1347447

[61] Biddle W, Schmitt MA, Fisk JD 2016 Modification of orthogonal tRNAs: unexpected consequences for sense codon reassignment. Nucleic Acids Res 44:10042–10050. doi:10.1093/nar/gkw948. 27915288 PMC5137457

[62] Mukai T, Lajoie MJ, Englert M, Söll D 2017 Rewriting the genetic code. Annu Rev Microbiol 71:557–577. doi:10.1146/annurev-micro-090816-093247. 28697669 PMC5772603

[63] Hammerling MJ, Gollihar J, Mortensen C, Alnahhas RN, Ellington AD, Barrick JE 2016 Expanded genetic codes create new mutational routes to rifampicin resistance in Escherichia coli. Mol Biol Evol 33:2054–2063. doi:10.1093/molbev/msw094. 27189550

[64] Ho JM, Reynolds NM, Rivera K, Connolly M, Guo LT, Ling J, Pappin DJ, Church GM, Söll D 2016 Efficient reassignment of a frequent serine codon in wild-type Escherichia coli. ACS Synth Biol 5:163–171. doi:10.1021/acssynbio.5b00197. 26544153 PMC4807657

[65] Chin JW 2014 Expanding and reprogramming the genetic code of cells and animals. Annu Rev Biochem 83:379–408. doi:10.1146/annurev-biochem-060713-035737. 24555827

[66] Liu CC, Schultz PG 2010 Adding new chemistries to the genetic code. Annu Rev Biochem 79:413–444. doi:10.1146/annurev.biochem.052308.105824. 20307192

[67] Carr PA, Church GM 2009 Genome engineering. Nat Biotechnol 27:1151–1162. doi:10.1038/nbt.1590. 20010598

[68] Kosuri S, Church GM 2014 Large-scale de novo DNA synthesis: technologies and applications. Nat Methods 11:499–507. doi:10.1038/nmeth.2918. 24781323 PMC7098426

[69] Gibson DG, Glass JI, Lartigue C, Noskov VN, Chuang RY, Algire MA, Benders GA, Montague MG, Ma L, Moodie MM, Merryman C, Vashee S, Krishnakumar R, Assad-Garcia N, Andrews-Pfannkoch C, Denisova EA, Young L, Qi ZQ, Segall-Shapiro TH, Calvey CH, Parmar PP, Hutchison CA, Smith HO, Venter JC 2010 Creation of a bacterial cell controlled by a chemically synthesized genome. Science 329:52–56. doi:10.1126/science.1190719. 20488990

[70] Hutchison CA, Chuang RY, Noskov VN, Assad-Garcia N, Deerinck TJ, Ellisman MH, Gill J, Kannan K, Karas BJ, Ma L, Pelletier JF, Qi ZQ, Richter RA, Strychalski EA, Sun L, Suzuki Y, Tsvetanova B, Wise KS, Smith HO, Glass JI, Merryman C, Gibson DG, Venter JC 2016 Design and synthesis of a minimal bacterial genome. Science 351:aad6253. doi:10.1126/science.aad6253. 27013737

[71] Smith HO, Hutchison CA, Pfannkoch C, Venter JC 2003 Generating a synthetic genome by whole genome assembly: φX174 bacteriophage from synthetic oligonucleotides. Proc Natl Acad Sci U S A 100:15440–15445. doi:10.1073/pnas.2237126100. 14657399 PMC307586

[72] Gibson DG, Smith HO, Hutchison CA III, Venter JC, Merryman C 2010 Chemical synthesis of the mouse mitochondrial genome. Nat Methods 7:901–903. doi:10.1038/nmeth.1515. 20935651

[73] Annaluru N, Muller H, Mitchell LA, Ramalingam S, Stracquadanio G, Richardson SM, Dymond JS, Kuang Z, Scheifele LZ, Cooper EM, Cai Y, Zeller K, Agmon N, Han JS, Hadjithomas M, Tullman J, Caravelli K, Cirelli K, Guo Z, London V, Yeluru A, Murugan S, Kandavelou K, Agier N, Fischer G, Yang K, Martin JA, Bilgel M, Bohutski P, Boulier KM, Capaldo BJ, Chang J, Charoen K, Choi WJ, Deng P, DiCarlo JE, Doong J, Dunn J, Feinberg JI, Fernandez C, Floria CE, Gladowski D, Hadidi P, Ishizuka I, Jabbari J, Lau CYL, Lee PA, Li S, Lin D, Linder ME, et al. 2014 Total synthesis of a functional designer eukaryotic chromosome. Science 344:55–58. doi:10.1126/science.1249252. 24674868 PMC4033833

[74] Boeke JD, Church G, Hessel A, Kelley NJ, Arkin A, Cai Y, Carlson R, Chakravarti A, Cornish VW, Holt L, Isaacs FJ, Kuiken T, Lajoie M, Lessor T, Lunshof J, Maurano MT, Mitchell LA, Rine J, Rosser S, Sanjana NE, Silver PA, Valle D, Wang H, Way JC, Yang L 2016 The genome project-write. Science 353:126–127. doi:10.1126/science.aaf6850. 27256881

[75] Wannier TM, Kunjapur AM, Rice DP, McDonald MJ, Desai MM, Church GM 2017 Long-term adaptive evolution of genomically recoded Escherichia coli. bioRxiv doi:10.1101/162834. PMC5866557 29440500

[76] Lenski RE, Barrick JE, Ofria C 2006 Balancing robustness and evolvability. PLoS Biol 4:e428. doi:10.1371/journal.pbio.0040428. 17238277 PMC1750925

[77] Masel J, Trotter MV 2010 Robustness and evolvability. Trends Genet 26:406–414. doi:10.1016/j.tig.2010.06.002. 20598394 PMC3198833

[78] Lee H, Popodi E, Tang H, Foster PL 2012 Rate and molecular spectrum of spontaneous mutations in the bacterium Escherichia coli as determined by whole-genome sequencing. Proc Natl Acad Sci U S A 109:E2774–E2783. doi:10.1073/pnas.1210309109. 22991466 PMC3478608

[79] Ruan B, Palioura S, Sabina J, Marvin-Guy L, Kochhar S, Larossa RA, Söll D 2008 Quality control despite mistranslation caused by an ambiguous genetic code. Proc Natl Acad Sci U S A 105:16502–16507. doi:10.1073/pnas.0809179105. 18946032 PMC2575449

[80] Mikkola R, Kurland CG 1992 Selection of laboratory wild-type phenotype from natural isolates of Escherichia coli in chemostats. Mol Biol Evol 9:394–402. 1584010 10.1093/oxfordjournals.molbev.a040731

[81] Netzer N, Goodenbour JM, David A, Dittmar KA, Jones RB, Schneider JR, Boone D, Eves EM, Rosner MR, Gibbs JS, Embry A, Dolan B, Das S, Hickman HD, Berglund P, Bennink JR, Yewdell JW, Pan T 2009 Innate immune and chemically triggered oxidative stress modifies translational fidelity. Nature 462:522–526. doi:10.1038/nature08576. 19940929 PMC2785853

[82] Meyerovich M, Mamou G, Ben-Yehuda S 2010 Visualizing high error levels during gene expression in living bacterial cells. Proc Natl Acad Sci U S A 107:11543–11548. doi:10.1073/pnas.0912989107. 20534550 PMC2895060

[83] Ribas de Pouplana L, Santos MAS, Zhu JH, Farabaugh PJ, Javid B 2014 Protein mistranslation: friend or foe? Trends Biochem Sci 39:355–362. doi:10.1016/j.tibs.2014.06.002. 25023410

[84] Lajoie MJ, Rovner AJ, Goodman DB, Aerni HR, Haimovich AD, Kuznetsov G, Mercer JA, Wang HH, Carr PA, Mosberg JA, Rohland N, Schultz PG, Jacobson JM, Rinehart J, Church GM, Isaacs FJ 2013 Genomically recoded organisms expand biological functions. Science 342:357–360. doi:10.1126/science.1241459. 24136966 PMC4924538

[85] Simon AJ, Ellington AD 2016 Recent advances in synthetic biosafety. F1000Res 5. doi:10.12688/f1000research.8365.1. PMC5007755 27635235

[86] Shaw AJ, Lam FH, Hamilton M, Consiglio A, MacEwen K, Brevnova EE, Greenhagen E, LaTouf WG, South CR, van Dijken H, Stephanopoulos G 2016 Metabolic engineering of microbial competitive advantage for industrial fermentation processes. Science 353:583–586. doi:10.1126/science.aaf6159. 27493184

[87] Mandell DJ, Lajoie MJ, Mee MT, Takeuchi R, Kuznetsov G, Norville JE, Gregg CJ, Stoddard BL, Church GM 2015 Biocontainment of genetically modified organisms by synthetic protein design. Nature 518:55–60. doi:10.1038/nature14121. 25607366 PMC4422498

[88] Rovner AJ, Haimovich AD, Katz SR, Li Z, Grome MW, Gassaway BM, Amiram M, Patel JR, Gallagher RR, Rinehart J, Isaacs FJ 2015 Recoded organisms engineered to depend on synthetic amino acids. Nature 518:89–93. doi:10.1038/nature14095. 25607356 PMC4590768

[89] Lorenz R, Bernhart SH, Höner Zu Siederdissen C, Tafer H, Flamm C, Stadler PF, Hofacker IL 2011 ViennaRNA package 2.0. Algorithms Mol Biol 6:26. doi:10.1186/1748-7188-6-26. 22115189 PMC3319429

[90] Kudla G, Murray AW, Tollervey D, Plotkin JB 2009 Coding-sequence determinants of gene expression in Escherichia coli. Science 324:255–258. doi:10.1126/science.1170160. 19359587 PMC3902468

